# Rationale for cannabis-based interventions in the opioid overdose crisis

**DOI:** 10.1186/s12954-017-0183-9

**Published:** 2017-08-18

**Authors:** Philippe Lucas

**Affiliations:** 1 Graduate Researcher, Centre for Addictions Research of British Columbia, 2300 McKenzie Ave, Victoria, BC V8N 5M8 Canada; 2 Social Dimensions of Health, University of Victoria, 3800 Finnerty Rd, Victoria, BC V8P 5C2 Canada; 3 VP, Patient Research & Access, Tilray, 1100 Maughan Rd, Nanaimo, BC V9X1J2 Canada

**Keywords:** Addiction, Opioids, Cannabis, Marijuana, Substitution, Harm reduction

## Abstract

**Background:**

North America is currently in the grips of a crisis rooted in the use of licit and illicit opioid-based analgesics. Drug overdose is the leading cause of accidental death in Canada and the US, and the growing toll of opioid-related morbidity and mortality requires a diversity of novel therapeutic and harm reduction-based interventions. Research suggests that increasing adult access to both medical and recreational cannabis has significant positive impacts on public health and safety as a result of *substitution effect*. Observational and epidemiological studies have found that medical cannabis programs are associated with a reduction in the use of opioids and associated morbidity and mortality.

**Aims and Methods:**

This paper presents an evidence-based rationale for cannabis-based interventions in the opioid overdose crisis informed by research on *substitution effect,* proposing three important windows of opportunity for cannabis for therapeutic purposes (CTP) to play a role in reducing opioid use and interrupting the cycle towards opioid use disorder: 1) prior to opioid introduction in the treatment of chronic pain; 2) as an opioid reduction strategy for those patients already using opioids; and 3) as an adjunct therapy to methadone or suboxone treatment in order to increase treatment success rates. The commentary explores potential obstacles and limitations to these proposed interventions, and as well as strategies to monitor their impact on public health and safety.

**Conclusion:**

The growing body of research supporting the medical use of cannabis as an adjunct or substitute for opioids creates an evidence-based rationale for governments, health care providers, and academic researchers to consider the implementation and assessment of cannabis-based interventions in the opioid crisis.

## Background

North America is currently in the grips of a crisis rooted in the use of licit and illicit opioid-based analgesics. Drug overdose is the leading cause of accidental death in Canada and the US, with many of these deaths amongst people affected by opioid use disorder. In 2015, there were 52,404 drug overdose deaths in the US, including 33,091 (63.1%) overdose deaths related to opioids [1]. In British Columbia, despite the declaration of a public health emergency in 2016 and the scale-up of public health-based efforts such as the opening of emergency overdose prevention sites in many high-use jurisdictions, use and overdose rates continue to rise. On April 26th British Columbia reported 130 opioid-related overdoses emergency calls in a single day,^1^ and in March 2017, 120 individuals died of drug overdoses.^2^ In light of the growing toll of opioid-based morbidity and mortality, this crisis requires a diversity of novel therapeutic and harm reduction-based interventions, and evidence suggests cannabis may have a role to play in reducing some of these harms.

### Substitution effect

Substitution effect is a theory originating from behavioral economics that examines how the availability of one good can impact and influence the use of other goods. In regards to substance use, Hursh et al. (2005) suggest that “pharmacological therapies for the treatment of drug abuse can also be conceptualized as alternative commodities that either substitute for illicit drug use (e.g., agonist therapy) or reduce the potency of illicit drugs directly (e.g., narcotic antagonist therapy)” [2]. Common examples of such harm reduction-focused substitution effect include the use of e-cigs or nicotine patches as alternatives to cigarettes, or methadone/suboxone treatment as an alternative to heroin. This paper presents an evidence-based rationale for cannabis-based interventions in the opioid overdose crisis informed by research on *substitution effect* and the principles of harm reduction.

There is a growing amount of evidence that increasing adult access to both medical and recreational cannabis has significant positive impacts on public health and safety, largely as a result of substitution effect. Population-level research describes how the introduction of regimes for legal access to cannabis (e.g., medical and/or recreational) in some US states has preceded reductions in homicides and violent crime [3], suicides [4], and automobile-related fatalities [5–7], all potentially related to subsequent declines in alcohol use. Additionally, epidemiological research has found that medical cannabis programs are associated with a reduction in the use of opioids and associated morbidity and mortality. Bachhuber et al. [8] report that U.S. states with medical cannabis laws had a 24.8% lower mean annual opioid overdose mortality rate compared to states without medical cannabis laws, and a 2016 study found that the number of Medicare prescriptions to seniors in medical cannabis states dropped for drugs that treat pain, depression, anxiety, nausea, psychoses, seizures and sleep disorders [9]. For pain, the annual number of annual doses prescribed per physician fell by 1826 doses. More recently, a retrospective survey of Michigan patients concluded that medical cannabis use was associated with a 64% decrease in opioid use (*n* = 118), decreased side effects of medications, and an improved quality of life [10], and a large survey of 2897 medical cannabis patients in California found that 30% of the sample (*n* = 841) reported using opioid-based pain medications, 97% of which “strongly agreed/agreed” that they were able to decrease their opioid use when using medical cannabis [11].

A 2015 cross sectional survey of patients in Canada’s national medical cannabis system found that 63% of respondents reported substituting cannabis for prescription drugs (*n* = 166), with 32% of the pharmaceuticals being substituted for being prescription opioids (*n* = 80). The primary reasons cited by patients for this substitution were “less adverse side effect” (39%, *n* = 68); “cannabis is safer” (27%, *n* = 48), and “better symptom management” (16%, *n* = 28) [12]. This evidence is consistent with information from Veteran’s Affairs Canada (VAC) showing that a recent significant increase in the use of medical cannabis by Canadian veterans was paralleled by a reduction of approximately 30% in the number of prescriptions for benzodiazepines, and a 16% decrease in the use of opioids [13].

Research suggests that there are multiple mechanisms of action that may result in the substitution of cannabis for opioids. In a study of cannabinoid-opioid interactions, Abrams et al. (2011) note that cannabinoids and opioids share many similar therapeutic and pharmacodynamics properties, including analgesic effects; the potential to induce hypothermia, sedation, and hypotension; as well as inhibition of intestinal motility and locomotor activity [14], adding that

“Synergy in analgesic effects between opioids and cannabinoids has been demonstrated in animal models. The antinociceptive effects of morphine are mediated predominantly by mu opioid receptors but may be enhanced by delta-9-tetrahydrocannabinol (THC) activation of kappa and delta opiate receptors [15]. It has further been suggested that the cannabinoid–opioid interaction may occur at the level of their signal transduction mechanisms [16, 17]. Receptors for both classes of drugs are coupled to similar intracellular signaling mechanisms that lead to a decrease in cyclic adenosine monophosphate production via G protein activation [17–19]. There is also some evidence that cannabinoids increase the synthesis and/or release of endogenous opioids.” (p. 844)

In light of the growing overdose crisis in North America, these findings on cannabis substitution effect and the biological mechanisms behind it strongly suggest that cannabis could play a role in reducing the public health impacts of prescription and non-prescription opioids. However, interventions testing the harm reduction potential of cannabis substitution effect have been lacking thus far. The following framework describes how novel cannabis-based interventions could minimize the personal and social harms associated with opioids.

## Methods

A compelling amount of evidence suggests there may be specific windows of opportunity for cannabis for therapeutic purposes (CTP) to play a role in the opioid use and dependence cycle. This commentary synthesizes the growing amount of research on cannabis substitution effect into specific policy recommendations aimed at improving public health and safety outcomes, with a focus on the 3 primary opportunities for cannabis to potentially reduce opioid use disorder and associated morbidity and mortality: 1) prior to opioid introduction in the treatment of chronic pain; 2) as an opioid reduction strategy for those already using opioids; and 3) as an adjunct therapy to methadone or Suboxone treatment in order to increase treatment success rates.

### Introduction/initiation

The pathway to opioid use disorder typically begins with the use of pharmaceutical opioids. Research suggests that 4 out of 5 heroin users report their opioid use began with prescription opioids [20]. If physicians and patients have access to a safer, less addictive alternative for pain control like cannabis [21], introducing it into the course of care as a first line treatment could potentially prevent the opioid overuse cycle from starting by not only reducing the risk pain patients would have of developing opioid use disorders, but also by reducing the overall supply of pharmaceutical opioids on the black market.

Clinical research on cannabis as a treatment for pain is extensive and suggests a relatively safe and effective treatment option [14, 22–26], and there is significant population-level evidence that cannabis substitution for opioids in the treatment of chronic pain is already taking place throughout North America. Chronic pain is the most common indication reported by Canadian and US patients who use medical cannabis [10, 27], and epidemiological studies by Bachhuber et al. [8] and Bradford and Bradford [9] strongly suggest that access to medical cannabis through state-level programs in the US reduces opioid use and related harms.

In light of this data, it would seem logical to seek to develop policies and associated education strategies to increase physician support for CTP in the treatment of chronic pain, and thereby reduce the health care provider community’s dependence on opioids as first or second line treatments options. However, while opioids remain second line treatment options throughout North America, clinical guidelines in Canada designate cannabis a third or fourth line treatment option for pain, and in the US, federal prohibition on the medical use of cannabis means that in many states, this is not an available treatment option under any circumstance.

It has become apparent that Canadian clinical guidelines and the US’s national prohibitionist policies are no longer reflective of the most current evidence and best available science on cannabis, opioids, and the treatment of chronic pain, and may in fact be inadvertently contributing to the growing rate of opioid use disorder. The growing body of research on the impact of cannabis on the use of other, potentially more dangerous substances creates a strong rationale to review these policies through a public health centered lens informed by the ongoing and increasing detrimental impacts of the current opioid crisis.

The argument in favor of recognizing medical cannabis as a first line option in the treatment of chronic pain is informed by science, common sense, and simple compassion: if patients never start using opioids, there is no risk their use might progress to dependence or overdose.

### Reduction/substitution

For those patients that are already using opioids in their course of care, the therapeutic imperative is to ensure treatment success without a progression to dependence and/or overuse. Evidence suggests that cannabis can be a useful adjunct therapy in meeting these goals. Cannabis augments the pain relieving potential of opioids [14], and can re-potentiate their effects [28], thereby reducing the need to increase the dosage of opioid pain medications. As noted earlier, cross-sectional and population-level research has shown that introducing cannabis into the treatment of chronic pain may result in a reduction or complete cessation of opioid use [11, 12, 29–33], thereby significantly reducing the potential for dependence or overdose. These findings suggest an opportunity to reduce opioid use through the development of therapeutic guidelines to safely introduce medical cannabis as an adjunct therapy for patients using opioids in the treatment of chronic pain. The aim of this strategy would be to slowly introduce cannabis into the continuum of care, while subsequently reducing the dosage and frequency of prescription opioid use.

However, here too there are some possible obstacles to implementation. Many members of the health care community and their respective organizations have expressed concerns about the use of medical cannabis, with much of the focus centering on smoking as a mode of use, and the impact of cannabis use on potentially vulnerable populations.

In regards to concerns over smoking as a route of administration, research suggests those who smoke cannabis regularly may be at increased risk of bronchial issues, however no causal link between cannabis use and lung or upper respiratory cancer has ever been established [34]. Encouragingly, recent patient surveys have found that alternatives to smoking such as vaporization and edibles are increasingly popular amongst patient and recreational populations [35, 36], and a 2015 survey of Canadian medical cannabis patients found that over 50% of patients report non-smoked options as their primary method of use [12]. Additionally, in Canada the availability of high quality oil-based extracts (e.g., drops and capsules) through the federal Access to Cannabis for Medical Purposes Regulations (ACMPR) provides patients and health care practitioners with legal, standardized alternatives to smoked ingestion. However, any cannabis-based medical intervention should be coupled with an educational campaign to discourage smoking and inform patients and physicians of safer alternative methods of use.

In regards to vulnerable populations, it’s certainly true that due to circumstances or pre-existing medical conditions, so individuals may not be well suited for cannabis-based therapies. In particular, a recent systematic review of medical cannabis and mental health suggests that “CTP users with psychotic disorders, and those at increased genetic risk of developing such disorders, should be cautioned regarding the use of cannabis” [37]. However, the same review also noted that medical cannabis may be useful in the treatment of post-traumatic stress disorder (often a co-morbidity with substance use issues), and that its use is not associated with increased violence. In fact, a 2014 study found that cannabis use resulted in reductions in interpersonal violence amongst married couples [38].

Other potentially vulnerable populations include youth and women who may be pregnant, and as with many currently available prescription drugs – including opioids - physicians should carefully weigh the potential harms and benefits of cannabis treatment when treating these populations.

Additionally, cannabis that is high in cannabidiol (CBD) and low in tetrahydrocannabinol (THC) may reduce potential harms to vulnerable populations. CBD is a relatively safe, non-impairing cannabinoid that has been shown to have many therapeutic effects relevant to the opioid crisis, including the reduction of heroin-seeking behavior in mice [39], and positive effects on mental health conditions like anxiety, depression, psychosis and bi-polar disorder [37, 40]. In other words, the existence of vulnerable populations should not result in abandoning or otherwise withholding this treatment option from others who might benefit from CTP, particularly in the treatment of chronic pain. It does however highlight the need to target outreach and education campaigns and specific treatment modalities aimed at reducing potential cannabis-related harms to these vulnerable populations.

### Replacement/Cessation

When opioid use graduates to dependence, it is imperative that users seeking opioid replacement therapy (ORT) enjoy the best possible chance of success, and some research has found that cannabis use can positively impact treatment success rates. For example, intermittent cannabis users showed superior retention in naltrexone treatment compared to abstinent or consistent users [41]. Additionally, objective ratings of opioid withdrawal decreased in patients concurrently using cannabis during the early stages of methadone stabilization [42], and CBD has been shown to reduce heroin seeking behavior in mice [39].

Greater ORT success rates reduce the risk of those with opioid use disorder suffering a relapse and subsequent fatal overdose, thereby diminishing the health care and public safety cost burden for all members of society. Since there is an exceedingly high risk of relapse and overdose in this dependent population [42, 43] - particularly with introduction of fentanyl and other powerful opioids into the illicit drug market - systematic research-based strategies to explore the potential of medical cannabis to improve ORT success rates should be implemented immediately. In order to address the need for good longitudinal data on the impact of cannabis-based medicines on methadone/Suboxone treatment, I have worked with Dr. Peter Farago to develop a multi-site cohort study that will compare the success rate of ORT in 250 cannabis using patients vs. 250 non-cannabis using controls. The study received ethics approval in May 2017 and will launch summer/fall 2017.

Patients seeking treatment for opioid use disorder deserve the best possible chance of success. Since evidence suggests that cannabis can help reduce opioid cravings and subsequently improve treatment retention and compliance, there is a strong rationale to immediately proceed with this novel intervention and associated studies.

### Implementation and assessment

It is notable that many of the favorable cannabis-related public health outcomes cited in this commentary did not come about as a result of a deliberate strategy to substitute cannabis for opioids, but rather through unintentional in situ changes in patient behavior resulting from cannabis use. This strongly suggests that a more purposeful and strategic approach to cannabis substitution for opioids may lead to even more encouraging outcomes, and Canada may be particularly well positioned to implement these proposed interventions. With a long-standing federally regulated medical cannabis program that currently serves over 150,000 Canadians with physician support for medical cannabis, and access to quality-tested medical cannabis products labeled for THC and CBD content, outreach and education to health care practitioners touting the three opportunities for cannabis-based interventions could be accomplished very quickly, and could thereby have nearly immediate impacts on opioid use.

Of interest in regards to the assessment and evaluation of these public policy measures, a number of provinces have centralized tracking of prescription drug dispensing, so detailed real-time data on the use of prescription opioids would be available to measure the population-level impacts of these interventions. This data could be coupled with well-designed epidemiological studies tracking overdose rates through first responder calls and emergency room data, as well as prospective observational cohort studies comparing methadone/suboxone treatment success rates in cannabis and non-cannabis using populations.

Observational and epidemiological research would not replace the need for high quality clinical trials examining the impact of cannabis on chronic pain, opioid use, and quality of life. Well-designed clinical trials continue to be necessary studies to determine the most effective method of use (inhalation or oral ingestion), optimal chemical composition (THC and CBD ratios and overall potency), and associated dosage to most effectively impact opioid use in all 3 of the proposed interventions. However, the significant public health impact of the current opioid crisis merits a rapid response strategy, and Canada’s federal Access to Cannabis for Medical Purposes Regulations and associated supply of cannabis and cannabis-based medications would allow for rapid implementation in a responsible and reflexive manner informed by existing regional, provincial and national pharmacovigilance and outcome assessment programs.

## Conclusion

Bureaucratic, legal and ideological obstacles to these interventions unquestionably exist in some jurisdictions. However, t is encouraging to see acknowledgements of the potential impacts of medical cannabis on opioid use from traditionally conservative organizations like the National Institute on Drug Abuse (NIDA), which recently acknowledged the growing scientific support for substitution effect on its website, noting that while “research into the effects of cannabis on opioid use in pain patients is limited…data suggest that medical cannabis treatment may reduce the dose of opioids required for pain relief”.^3^

Cannabis alone will not end opioid use disorder and associated morbidities and mortality. However, the introduction of ever more powerful opioids like fentanyl and carfentanyl into the illicit drug market and the resulting day-to-day increase in opioid overdoses highlights the immediate need for innovative short and long term intervention strategies to add to current efforts like ORT, heroin maintenance programs, supervised consumption sites, ﻿the depenalization of substance use,﻿ and increased education and outreach on the potential harms associated with both prescription and illicit opioid use. The growing body of research supporting the medical use of cannabis as an adjunct or substitute for opioids creates an evidence-based rationale for governments, health care providers, and academic researchers to seek the immediate implementation of cannabis-based interventions in the opioid crisis at the regional and national level, and to subsequently assess their potential impacts on public health and safety.

## Footnotes

1 Matt Meuse, April 27, 2017. B.C. breaks record for daily overdose ambulance calls. CBC News.

2 Staff, April 19th, 2,017,120 died in B.C. last month of illicit overdoses. Global News. http://globalnews.ca/news/3389500/120-died-in-b-c-last-month-of-illicit-overdoses/

3
https://www.drugabuse.gov/publications/marijuana/marijuana-safe-effective-medicine
